# Association of ABO blood group with severe falciparum malaria in adults: case control study and meta-analysis

**DOI:** 10.1186/1475-2875-10-309

**Published:** 2011-10-19

**Authors:** Aditya K Panda, Santosh K Panda, Aditya N Sahu, Rina Tripathy, Balachandran Ravindran, Bidyut K Das

**Affiliations:** 1 Infectious Disease Biology Group, Institute of Life Sciences, Bhubaneswar, Odisha, India; 2 Department of Medicine, S.C.B. Medical College, Cuttack, Odisha, India; 3 Department of Biochemistry, S.C.B. Medical College, Cuttack, Odisha, India

**Keywords:** ABO blood group, severe malaria, cerebral malaria, multi-organ dysfunction, non-cerebral severe malaria, uncomplicated malaria, meta-analysis

## Abstract

**Background:**

Erythrocyte-associated antigenic polymorphisms or their absence have perhaps evolved in the human population to protect against malarial infection. Studies in various populations consistently demonstrate that blood group 'O' confers resistance against severe falciparum infection. In India, Odisha state has one of the highest incidences of *Plasmodium falciparum *infection and contributes to the highest number of deaths by falciparum malaria. This study aims to evaluate the relationship between ABO blood group and severe malaria in an adult population at the tertiary care centre in Odisha.

**Methods:**

A total of 353 *P. falciparum *infected subjects and 174 healthy controls were screened for ABO blood group. Falciparum-infected individuals were categorized as severe malaria and uncomplicated malaria. Severe malaria was further clinically phenotyped into cerebral malaria, non-cerebral severe malaria and multi-organ dysfunction. A meta-analysis was performed to assess the role of ABO blood group in severe malaria.

**Results:**

Frequency of blood group 'B' was significantly higher in patients with severe malaria compared to the uncomplicated cases (P < 0.0001; OR = 4.09) and healthy controls (P < 0.0001; OR = 2.79). Irrespective of the level of clinical severity, blood group 'B' was significantly associated with cerebral malaria (P < 0.0001; OR = 5.95), multi-organ dysfunction (P < 0.0001; OR = 4.81) and non-cerebral severe malaria patients (P = 0.001; OR = 3.02) compared to the uncomplicated category. Prevalence of 'O' group in uncomplicated malaria (P < 0.0001; OR = 2.81) and healthy controls (P = 0.0003; OR = 2.16) was significantly high compared to severe malaria. Meta-analysis of previous studies, including the current one, highlighted the protective nature of blood group 'O' to severe malaria (P = 0.01). On the other hand, carriers of blood group 'A' (P = 0.04) and 'AB' (P = 0.04) were susceptible to malaria severity.

**Conclusions:**

Results of the current study indicate that blood group 'O' is associated with reduced and 'B' blood group with increased risk of development of severe malaria in Odisha, India. Meta-analysis also supports the protective nature of blood group 'O' from severe falciparum infection.

## Background

Malaria is an infection caused by protozoan parasites of the genus *Plasmodium *and transmitted by the bite of infected *Anopheles *mosquitoes. Out of the four species that infect humans, *Plasmodium falciparum *is the principal cause of severe clinical manifestations [1]. Cyto-adherence and rosetting are important components of several possible pathogenic mechanisms attributed to the cause of severe infection [2]. An association between 'O' blood group and lower rosetting capacity has been demonstrated [3]. However, rosetting capacities of blood group 'A', 'B' or 'AB' have remained controversial [4-7]. On the erythrocyte surface, the A and B antigens are tri-saccharides -A, GalNAcα1-3(Fucα1-2)Gal1β1; and B- Gal1α1-3(Fucα1-2)Galβ1 respectively, that are attached to different glycolipids and glycoproteins [8]. An enzyme glucotransferase is necessary for the production of A and B antigens. On the other hand, blood group 'O' carries a disaccharide H antigen (Fucα1-2Galβ1) due to the absence of the enzyme glucotransferase [8]. Variations in gene encoding functional glucotransferase have been associated with protections from severe *P. falciparum *malaria [9] and this observation has been further strengthened by a recent genome wide association study [10]. Tri-saccharide of 'A' and 'B' blood group is presumed to act as receptors and functions as an important factor for rosetting [7]. However, RBCs of blood group 'O' do not express tri-saccharide, and rosettes formed by infected 'O' blood group RBCs are smaller and easily disrupted compared to blood groups A, B or AB [4,7,11].

There are limited reports in literature on association of ABO blood group and susceptibility to severe falciparum malaria.. The association of blood group 'AB' and severe malaria has been demonstrated in various populations, viz. Sri Lanka [12], Mali [11], and Ethiopia [1], while a significant association has also been reported between blood group 'A' and severity in Gabon [13], Ethiopia [1] and Zimbabwe [14]. The role of blood group 'B' and severe falciparum infection has not been reported.

Malaria remains a major health problem in India. The National Vector Borne Disease Control Programme (NVBDCP), India, has reported that 1.8 million cases of malaria and 1,000 malaria-related deaths occur annually [15]. However, the World Health Organization (WHO) estimates that figure to be 20 million cases and 15,000 deaths [16]. A recent study reported a staggering 1,22,000 deaths due to malaria in India, and Odisha as a major contributor to this mortality [17]. Although the state is hyper-endemic to *P. falciparum malaria *[18] and contributes 29.8% of deaths related to the infection [15], no study has been carried out in the local population to assess the association of ABO blood group in severe infection. Therefore, the current study aims to investigate this association and look into the overall role of ABO blood group in risk/resistance to the development of severe malaria by a meta-analysis of results from the current study and earlier published reports.

## Methods

### Study site and participants

The study was conducted at S.C.B. Medical College, Cuttack, Odisha, India between 2008-2009. Patients (age ≥15 years) admitted to the Department of Medicine with a short history of fever were clinically examined in detail and screened for *P. falciparum *infection by Giemsa-stained thick and thin blood smears and immune chromatography test (SD Bio Standard Diagnostics India). Detection of *P. falciparum *was also performed by nested polymerase chain reaction (PCR). Individuals infected only with *P. falciparum *were included. Clinical categorization was done based on WHO guidelines [19]. Uncomplicated malaria (UM) was defined as patients with fever and evidence of falciparum infection in the blood. Severe malaria(SM) was categorized into three groups based on distinct clinical features: 1) Cerebral malaria (CM), 2) Non cerebral severe malaria (NCSM) and 3) Multi-organ-dysfuction (MOD). CM was further defined as patients with altered sensorium, GCS (Glasgow Coma Scale) of ≤ 10. NCSM patients had one of the several manifestations of severe malaria without cerebral involvement, namely severe anaemia (haemoglobin <5 g/dl), acute renal failure (serum creatinine >3 mg/dl), jaundice (serum bilirubin >3 mg/dl), acute respiratory distress syndrome (PaO2/FIO2 <200), haemoglobinuria (dark red or black coloured urine positive for haemoglobin) and shock (systolic BP of <80 mm Hg). MOD was diagnosed based on presence of two or more organ involvement like CNS (GCS≤10), respiratory (PaO2/FIO2 <200), renal failure (serum creatinine >3 mg/dl) and hepatic dysfunction (ALT/AST >3 times of normal, prolonged prothrombin time and albuminaemia). 174 healthy controls (HC) of identical ethnicity and hailing from a similar geographical background were enrolled. None of the controls reported history of clinical malaria in the last 5 years. They were essentially healthy and negative for demonstrable *P. falciparum *infection. The risk of exposure to malaria was similar for both HC and patients. Criteria for analysis of mortality: Since death occurred in the CM and MOD groups' only patients from these groups were included for analysis. The study was approved by the Institutional Ethics Committee of the Medical College and blood samples were collected after written consent of the patients or accompanying person, depending on the clinical scenario.

### Polymerase chain reaction

Genomic DNA was extracted by GenElute™ Blood Genomic DNA Kit (SIGMA) from whole blood according to the manufacturer's instructions. Polymerase chain reaction (PCR), has been used to detect up to species level - falciparum, vivax, ovale and malariae [20,21]. In the population under study, *P. falciparum *is the major cause of malaria (>80%) followed by *P. vivax *(10-15%) [22]. With slight modification, genus and species specific nested PCR technique was used to detect *P. falcipaurm *and/or *P. vivax *infections [20]. In brief, primary PCR (to detect *Plasmodium *genus) was performed in a 20 μl PCR reaction containing 3 μL genomic DNA, 1× Taq buffer containg MgCl_2 _(SIGMA), 250 μM dNTP (SIGMA)), 200 nM of two genus-specific primer rPLU1 (5'-TCA AAG ATT AAG CCA TGA AAG TGA-3') and rPLU5 (5'-CCT GTT GTT GCC TTA AAC TCC-3') (Integrated DNA technologies) and 1 U of DNA polymerase (SIGMA). The cycle conditions were as follows: an initial de-naturation step at 95°C for 7 minutes, followed by 38 cycles of 95°C for 30 seconds, annealing at 55°C for 30 seconds, extension at 72°C for 1 minute, and a final extension at 72°C for 7 minutes. For secondary PCR (to detect falciparum and/or vivax species), 3 ul of PCR product of previous reaction was used as template and species-specific primers set were used {rFAL1(5'-TTA AAC TGG TTT GGG AAA ACC AAA ACC AAATAT ATT)/rFAL2(5'-ACA CAA TGA ACT CAA TCA TGA CTA CCC GTC-3') and rVIV1(5'-CGC TTC TAG CTT AAT CCA CAT AAC TGA TAC-3')/rVIV2(5'-ACT TCC AAG CCG AAG CAA AGA AAG TCC TTA-3')} in different tubes. Reaction mixture and thermo-cycler condition was similar to primary PCR. PCR products were analysed in agarose gel electrophoresis.

### ABO blood group typing

Blood samples of both falciparum-infected individuals and healthy controls were typed by commercial haemagglutination kit (Tulip Diagnostics, Goa, India). In brief, about 20 ul of whole blood was taken on a clean slide and 20 ul of antisera A, B and Rhesus blood group applied, mixed by means of an applicator stick and results were noted.

### Identification of studies for meta-analysis

An extensive PubMed search was performed for articles published on the association of ABO blood group in severe *P. falciparum *malaria. Cross-references were checked including studies not located in PubMed. The following key words and subject terms were searched: ABO blood group, *Plasmodium falciparum*, severe malaria and uncomplicated malaria.

### Statistical analysis

Statistical analyses were performed by using GraphPad Prism (version 5.01). Difference between means was analysed by ANOVA. The Fisher exact test was used to analyse the association between blood groups and severe malaria or different clinical manifestation. Odds ratio (OR) were calculated with 95% confidence interval. The 'O' blood group was taken as a reference to compare the prevalence of other blood groups, 'A', 'B', 'AB' and non 'O', defined as A+B+AB combined, in severe and uncomplicated malaria. A probability value of ≤ 0.01 (0.05/4) was considered statistically significant after Bonferroni correction. Meta-analysis and all related statistics were obtained by Comprehensive Meta-analysis V2 software. Variation within and among different study (heterogeneity) was assessed by Q-test with the null hypothesis that all studies have the same effects. The random effects model was used for meta-analysis if the Q-statistic was significant (P < 0.05) which indicate heterogeneity across studies. On the other hand, lineage of all studies to null hypothesis, the fixed effect model, was used for necessary meta-analysis.

## Results

### Characteristics of study participants

A total of 353 falciparum-infected patients were enrolled in the present study, including 247 severe (SM) and 106 uncomplicated patients (UM). Severe *P. falciparum *malaria patients were further subdivided into cerebral malaria (CM) (n = 59), multi-organ dysfunction (MOD) (n = 80) and non-cerebral severe malaria (NCSM) (n = 108). 174 healthy controls (HC) were included. The mean age between all clinical categories and HC was comparable. Significantly higher levels of haemoglobin were observed in HC than UM and other clinical categories of severe malaria (P < 0.0001) (Table 1).

**Table 1:** Details of study participants

Subjects	Severe *P. falciparum *malaria clinical categories (SM)			UM	HC	P value
	CM	MOD	NCSM			
**Total number (n)**	59	80	108	106	174	NA
**Sex (M/F)**	42/17	65/15	91/17	84/22	134/40	NA
**Mean age in years (range)**	33.5 (15-65)	33.5 (15-70)	34 (15-65)	32.5 (15-80)	31.1 (16-75)	NS
**Mean Hg (g/dl)±SD**	9.9 ± 1.9	9.9 ± 2.2	9.7 ± 2.5	10.82 ± 1.9	12.8 ± 1.9	< 0.0001

SM: severe malaria; CM: cerebral malaria; MOD: multi-organ dysfunction; NCSM: non-cerebral severe malaria; UM: uncomplicated malaria; HC: healthy control; NA: not applicable; NS: not significant

### Distribution of ABO blood group in severe malaria, uncomplicated malaria and healthy controls

To assess possible associations of ABO blood groups with severe course of *P. falciparum *infection, prevalence of each ABO blood group was compared in severe malaria patients (n = 247), uncomplicated malaria (n = 106) and healthy controls (n = 174). Of the 174 blood samples examined in healthy controls, 74 (42%), 36 (21%), 50 (29%) and 14 (8%) individuals were from O, A, B, and AB blood group, respectively. As shown in Table 2, prevalence of B blood group was significantly higher in severe malaria (SM) compared to UM (P < 0.0001; OR = 4.09) and HC (P < 0.0001; OR = 2.79). Furthermore, blood group 'O' was significantly associated with UM compared to the severe categories (P < 0.0001; OR = 2.81) indicating its likely protective role (Table 2). The distribution of blood groups among HC and UM was comparable.

**Table 2:** Prevalence of ABO blood group in different *P. falciparum *malaria clinical categories and healthy controls

Blood group	SM (n = 247)	UM (n = 106)	HC (n = 174)	SM vs UM P value, OR (95% CI)	SM vs HC P value, OR (95% CI)	UM vs HC P value, OR (95% CI)
**O**	63 (25)	52 (49)	74 (42)	ref	ref	ref
**A**	48 (19)	20 (19)	36 (21)	0.04, 0.50 (0.26 to 0.95)	0.12, 0.63 (0.36 to 1.10)	0.51, 1.26 (0.65 to 2.42)
**B**	119 (49)	24 (23)	50 (29)	<0.0001, 4.09 (2.30 to 7.25)	<0.0001, 2.79 (1.74 to 4.47)	0.23, 1.46 (0.80 to 2.67)
**AB**	17 (7)	10 (9)	14 (8)	0.52, 0.71 (0.30 to 1.69)	0.42, 0.70 (0.32 to 1.53)	1.00, 0.98 (0.40 to 2.38)
**Non O (A+B+AB)**	184 (75)	54 (51)	100 (58)	<0.0001, 2.81 (1.74 to 4.52)	0.0003, 2.16 (1.42 to 3.27)	0.32, 1.30 (0.80 to 2.11)

NOTE: Data are no. (%) of participants unless otherwise specified.

SM: severe malaria; UM: uncomplicated malaria; HC: healthy controls; OR: odds ratio; CI: confidence interval

### Prevalence of ABO blood group in various clinical phenotypes of severe *Plasmodium falciparum *malaria

The association of ABO blood group in different clinical phenotypes of severe malaria, categorized as CM, NCSM and MOD, was studied. As highlighted in Table 3, prevalence of blood group 'B' was significantly higher in CM (P < 0.0001; OR = 5.95), MOD (P < 0.0001; OR = 4.81) and NCSM (P = 0.001; OR = 3.02) compared to UM. Furthermore, the protective nature of group 'O' was observed when comparing its prevalence in UM vis-a-vis the different severe groups (CM: P = 0.0003; OR = 3.77, MOD: P = 0.0002; OR = 3.31 and NCSM: P = 0.007; OR = 2.81).

**Table 3:** Distribution of ABO blood group in different clinical subtypes of severe falciparum infections and uncomplicated malaria

Blood group	CM (n=59)	MOD (n=80)	NCSM (n=108)	UM (n=106)	CM vs UM P value, OR (95%CI)	MOD vs UM P value, OR (95%CI)	NCSM vs UM P value, OR (95%CI)
**O**	12 (20)	18 (22)	33 (31)	52 (49)	ref	ref	ref
**A**	7 (12)	19 (24)	22 (20)	20 (19)	0.57, 0.65 (0.22 to 1.91)	0.02, 0.36 (0.15 to 0.83)	0.18, 0.57 (0.27 to 1.21)
**B**	33 (56)	40 (50)	46 (43)	24 (23)	<0.0001, 5.95 (2.62 to 13.52)	<0.0001, 4.81 (2.30 to 10.06)	0.001, 3.02 (1.56 to 5.83)
**AB**	7 (12)	3 (3)	7 (6)	10 (9)	0.10, 0.32 (0.10 to 1.04)	1.00, 1.15 (0.28 to 4.66)	1.00, 0.90 (0.31 to 2.61)
**Non O (A+B+AB)**	47 (80)	62 (78)	75 (69)	54 (51)	0.0003, 3.77 (1.80 to 7.90)	0.0002, 3.31 (1.73 to 6.34)	0.0078, 2.81 (1.25 to 3.82)

NOTE: Data are no. (%) of participants unless otherwise specified.

CM: cerebral malaria; MOD: multi-organ dysfunction; NCSM: non-cerebral severe malaria; UM: uncomplicated malaria; OR: odds ratio; CI: confidence interval

### Blood group B and prognosis

Since the study revealed a significant association of ABO blood group and severe falciparum malaria, possible role of ABO blood groups in disease outcome was explored. Prevalence of blood groups were analysed among subjects who died during treatment and those who survived Out of 247 cases of severe malaria, twenty patients died during course of treatment: five patients from CM category (n = 59), 15 patients from MOD (n = 80) and none from NCSM (n = 108). Therefore, NCSM patients were not included in the analysis. Furthermore, fifteen patients from CM and MOD groups left the hospital against medical advice, which necessitated their exclusion since their survival status was not known. As a result, a total of 124 patients in CM and MOD groups were analysed. Although, frequency of blood group 'B' was higher in patients who died (80%) compared to those who survived (54%), the difference was not statistically significant (P = 0.06). Distribution of other blood groups in patients who died and survived was comparable (Table 4).

**Table 4:** Association of ABO blood group in treatment outcome patients with severe malaria

Blood group	Dead (n = 20)	Survivors (n = 104)	P value, OR (95%CI)
**O**	1 (5)	21 (20)	ref
**A**	2 (10)	18 (17)	0.59, 0.42 (0.03 to 5.12)
**B**	16 (80)	56 (54)	0.06, 6.00 (0.74 to 48.13)
**AB**	1 (5)	9 (9)	0.53, 0.42 (0.02 to 7.63)
**Non O (A+B+AB)**	19 (95)	83 (80)	0.12, 0.20 (0.02 to 1.64)

NOTE: Data are no. (%) of participants unless otherwise specified;

OR: odds ratio; CI: confidence interval

### Studies included in meta-analysis

Nineteen relevant publications were identified on falciparum malaria and ABO blood group. The three primary criteria for inclusion in this meta-analysis were as follows:1) study must be case-controlled, 2) patients placed under well defined categories as severe and uncomplicated, 3) the sample size should be more than 50 in each category. Out of nineteen studies only five publications satisfied all inclusion criteria and were considered for meta-analysis. Data from the recent study was also included for analysis. Table 5 shows characteristics of all six studies.

**Table 5:** Characteristics of individual studies summarized for the meta-analysis

Author, Year [Ref]	Population	Sample	Blood Groups			
			
			O	A	B	AB
**Al-Yaman *et al. *1995 **[34]	Papua New Guinea	SM (n = 97)	44 (45)	29 (30)	10 (10)	14 (15)
		
		UM (n = 156)	53 (34)	55 (35)	28 (18)	20 (13)

**Lell *et al. *1999 **[13]	Gabon	SM (n = 100)	54 (64)	27 (27)	Data not available	Data not available
		
		UM (n = 100)	64 (64)	11 (11)	Data not available	Data not available

**Pathirana *et al. *2005 **[12]	Sri Lanka	SM (n = 80)	19 (24)	26 (32.5)	22 (27.5)	13 (16)
		UM (n = 163)	78 (48)	40 (24)	37 (23)	8 (5)

**Rowe *et al. *2007 **[11]	Mali	SM (n = 124)	26 (21)	40 (32)	11 (9)	47 (38)
		
		UM (n = 124)	55 (44)	31 (25)	9 (7)	29 (24)

**Tekeste *et al. *2010 **[1]	Ethiopia	SM (n = 70)	16 (23)	25 (36)	15 (21)	14 (20)
		
		UM (n = 140)	64 (46)	42 (30)	23 (16)	11 (8)

**Current study**	India	SM (n = 247)	63 (25)	48 (19)	119 (49)	17 (7)
		
		UM (n = 106)	52 (49)	20 (19)	24 (23)	10 (9)

NOTE: Data are no. (%) of participants unless otherwise specified.

SM: severe malaria; UM: uncomplicated malaria;

### Evaluation of publication bias

A funnel plot has been used to test publication bias in meta-analysis. The funnel plot remains symmetric in absence of publication bia**s**. For 'O', 'B' and 'AB' blood groups the funnel plots were symmetric. However, plot for blood group 'A' was asymmetric. Trim-and-fill technique was used to adjust publication bias of blood group 'A', for recomputing the effect size [23].

### Association of ABO blood group and severe malaria

Heterogeneity Q test was performed to evaluate inter- and intra-study variations and based upon significance value; different models were used for the meta-analysis. Blood group 'O' (P < 0.0001), 'B' (P = 0.004) and 'AB' (P = 0.037) showed significant heterogeneity and, therefore, a random-affect model was employed. On the other hand, a fixed-effect model was used for association of blood group 'A' since the Q-test was not significant (P = 0.120).

**Figure 1:**
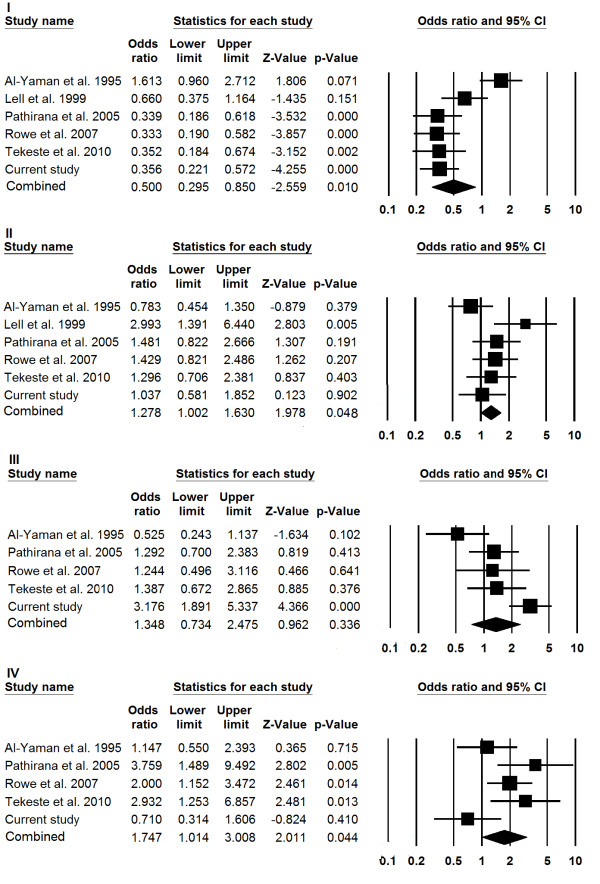
**Forest plots of blood groups in association to severe malaria**. Meta-analysis was performed including previous reports and current study by comprehensive meta-analysis software. Random or fixed model of meta-analysis was employed for calculation of the combined effect of all studies. Forrest plots evaluating resistance/risk factor of blood group O (I), A (II), B (III) and AB (IV) to severe malaria are shown.

Results of meta-analysis are shown in Figure 1. Protection against severe malaria was significantly associated with blood group 'O' (P = 0.01). In contrast, blood group 'A' and 'AB' showed significant association to susceptibility ('A': P = 0.04 and 'AB': P = 0.04). Although, 'B' blood group was significantly associated with severe malaria in the present study, the meta-analysis failed to corroborate that association.

## Discussion

Results of the current study suggest that patients with blood group 'B' have a four-fold increased risk of developing severe infection. In addition, it also reiterates the observation that 'O' blood group was significantly associated with a decreased risk of severe malaria. Other blood groups ('A' and 'AB') did not show any association.

The role of ABO blood group in malaria has been investigated in various populations, but robust data is limited [24]. This study was an attempt to analyse the association of ABO blood group in large number of adult patients and healthy controls in Odisha, a state, highly endemic for falciparum infection [18]. Higher prevalence of blood group 'O' was observed in uncomplicated cases, an indication of its possible protective property against severity as indicated in previous reports [1,11-13]. The mechanism of protection is not clearly understood. It is postulated that the phenomenon of rosetting is one of the mechanisms that contributes to disease severity [2]. This rosetting capacity varies among different blood groups [3]. Lowest rosette formation is observed in blood group 'O' individuals. This study also clearly highlights the association of severe malaria with blood group 'B'. Interestingly, this association was valid across all grades of severity. Although the number of patients who died (n = 20) was small, frequency of blood group 'B' was higher in these subjects (80%) in comparison to survivors (54%). Previously, studies on patients from Zimbabwe [14], Gabon [13] and Ethiopia [1] showed a significant association of 'A' blood group with severe malaria. Blood group 'AB' has also been reported to be associated with severity in Sri Lanka [12], Mali [11] and Ethiopian populations [1]. There are no reports implicating blood group 'B' with severity. The variability of observations made with regard to different blood groups may be attributable to different rosetting capacity, heterogenous population groups and varied infective strains [3]. Blood group 'A' in Uganda and Gambia [4,7], 'B' group in Thailand [4] and 'AB' group in Kenya [6] have been associated with increased rosetting phenomenon.

It is presumed that the prevalence of blood group 'O' would be higher in malaria endemic areas due to its capacity to confer protection. An analysis of blood group in healthy controls revealed a distribution of 'O' (42%), which was much higher compared to 'A' (21%), 'B' (29%) and 'AB' (8%). A community-based study in a tribal population of Odisha, where malaria is endemic, also showed higher prevalence of blood group 'O' [25]. Significantly, a lower prevalence 'O' blood group has been observed in other malaria non-endemic states like Maharashtra [26,27] and Uttar Pradesh [28] in India, indicating a selective advantage of this blood group in endemic localities. This hypothesis is further supported by higher prevalence of 'O' blood group worldwide where malaria infection is prevalent [29].

Meta-analysis combines results of several similar studies to produce a single estimate of the major effect with enhanced precision [30]. Current analysis revealed significant association of blood group 'O' with protection against severe malaria (OR = 0.45) In contrast, blood groups 'A' and 'AB' were associated with susceptibility to severity: Blood group 'A' and 'AB' conferred 1.27- and 1.74- fold higher risks respectively. Although in the current study, 'B' blood group was significantly associated with severe malaria on meta-analysis the association was insignificant. This variation may be a population specific phenomenon.

There are however limitations in the present study. Several RBC polymorphisms, including those linked to glucose-6-phosphate dehydrogenase, pyruvate kinase, complement receptor-1 and haemoglobinopathies, have a role in the clinical outcome of malaria [31], but were not included for analysis in the present study. Such polymorphisms have only been reported from the Western belt of Odisha among tribal communities [32,33] and not in areas from which patients and HC in the current study were enrolled.

## Conclusion

This study reveals a significant association of blood group 'B' to severe malaria. The association is valid across all grades of severity. Blood group 'O' confers protection to severe disease. The meta-analysis reiterates the observation of protection conferred by blood group 'O' and highlights susceptibility of group 'A and 'AB' to severe malarial infection.

## Competing interests

The authors declare that they have no competing interests.

## Authors' contributions

AKP was involved in detection of malaria by PCR, analysis, interpretation, performed statistics and writing the first draft of the manuscript. SKP was also involved in species-specific PCR and analysis. ANS, RT, BR and BKD made a contribution in the design, data interpretation, work supervision and critically revising the manuscript. All authors read and approved the manuscript.
